# Toward the Development of a Circulating Free DNA-Based *In Vitro* Diagnostic Test for Infectious Diseases: a Review of Evidence for Tuberculosis

**DOI:** 10.1128/JCM.01234-18

**Published:** 2019-03-28

**Authors:** B. Leticia Fernández-Carballo, Tobias Broger, Romain Wyss, Niaz Banaei, Claudia M. Denkinger

**Affiliations:** a FIND, Geneva, Switzerland; b Department of Pathology, Clinical Microbiology Laboratory, Stanford Health Care, Stanford, California, USA; c Department of Medicine, Clinical Microbiology Laboratory, Stanford Health Care, Stanford, California, USA; d Stanford School of Medicine, Clinical Microbiology Laboratory, Stanford Health Care, Stanford, California, USA; Emory University

**Keywords:** *Mycobacterium tuberculosis*, diagnosis, liquid biopsy

## Abstract

The detection of circulating free DNA (cfDNA) has transformed the field of oncology and prenatal diagnostics. Clinical application of cfDNA for disease diagnosis and monitoring, however, is relatively recent in the field of infectious disease. The potential of cfDNA as a noninvasive diagnostic and monitoring tool is especially promising for tuberculosis (TB), as it enables the detection of both pulmonary and extrapulmonary TB from easily accessible urine and/or blood samples from any age group. However, despite the potential of cfDNA detection to identify TB, very few studies are described in the literature to date. A comprehensive search of the literature identified 15 studies that report detecting Mycobacterium tuberculosis DNA in the blood and urine of TB patients with nongenitourinary disease, but in only six of them were the methodological steps considered suitable for cfDNA isolation and detection. The sensitivities and specificities for the diagnosis of pulmonary and extrapulmonary TB cases reported in these six studies are highly variable, falling in the range of 29% to 79% and 67% to 100%, respectively. While most studies could not meet the performance requirements of the high-priority target product profiles (TPP) published by the World Health Organization (WHO), the study results nonetheless show promise for a point-of-care detection assay. Better designed prospective studies, using appropriate samples, will be required to validate cfDNA as a TB biomarker.

## Abstract (precis)

The detection of circulating free DNA (cfDNA) has transformed the field of oncology and prenatal diagnostics. Clinical application of cfDNA for disease diagnosis and monitoring, however, is relatively recent in the field of infectious disease.

## INTRODUCTION

Circulating cell-free DNA (cfDNA) in human blood was first discovered in 1948 (1) but did not attract much interest for infectious disease diagnosis and monitoring until decades later, when technologies had evolved to harness the potential of cfDNA for a noninvasive, rapid, and sensitive approach to diagnosis.

cfDNA comprises fragments of nucleic acids found in the acellular fraction of blood and other biological fluids (2). These nucleic acids are believed to derive from dying human cells and microorganisms that release their contents into the blood as they break down. cfDNA is much smaller than genomic DNA and more than 70% of plasma cfDNA is smaller than 300 bp, with an average size of 170 bp (3–5). It is hypothesized that the small size allows cfDNA to cross the kidney barrier and appear in the urine (6). The cfDNA concentration in the blood of healthy individuals varies widely, from less than 10 ng/ml to more than 1,500 ng/ml, which corresponds to approximately 1,400 to 200,000 DNA copies/ml of a diploid human genome split into ∼170-bp fragments (assuming that the size of a diploid human genome is 6,469.66 Mbp and 650 dalton/bp) (5). Considerable differences in the size distribution and quantity of cfDNA in the urine and blood were described between different individuals and even within the same individual under certain disease conditions (e.g., cancer and infectious diseases) and physiological states (e.g., pregnancy) (2, 7).

The detection of cfDNA is currently used in a range of clinical applications to guide clinical decisions. The main areas where cfDNA is used include noninvasive prenatal testing, oncology, and transplantation (2, 7). In the cancer field, circulating tumor DNA (ctDNA) present in frequencies as low as 0.00025% of the total cfDNA in circulation is accurately detected using a targeted next-generation sequencing (NGS) approach (called cancer personalized profiling by deep sequencing) (8–11). Targeted techniques, including digital PCR (dPCR) and beads, emulsions, amplification, and magnetics (BEAMing), allow the detection of ctDNA present in frequencies in the range of 1% to 0.001% of the total cfDNA in circulation (8–11). Presumably, such detection limits should be achievable for other diseases as well.

cfDNA has also been applied for the diagnosis of infectious diseases. For several decades, the use of cfDNA has been reported for the detection of Epstein-Barr virus for nasopharyngeal carcinoma screening and more recently for the diagnosis of invasive fungal infection (12, 13). Some specific examples of infectious agents reported to be detected using cfDNA include *Plasmodium*, *Trypanosoma*, *Leishmania*, *Schistosoma*, *Leptospira*, and HIV (2, 14). Interest in cfDNA for the diagnosis of infectious diseases is growing, especially for those diseases and/or specific cases for which no appropriate tests on easily accessible samples (blood or urine) are available on the market.

**FIG 1:**
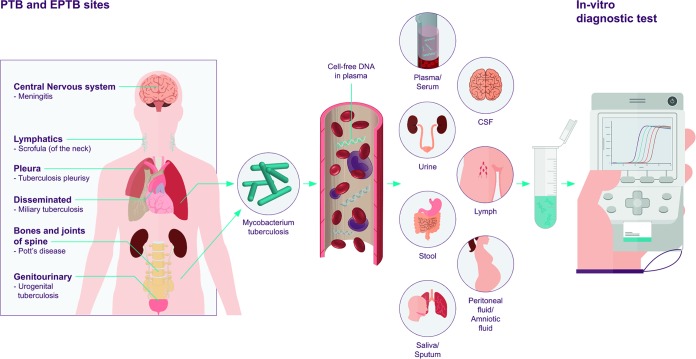
Schematic drawing of the origin, release, and potential diagnostic use of M. tuberculosis cfDNA within the human host. M. tuberculosis within the lungs or in extrapulmonary sites release cell-free DNA into the blood circulation, which then may be redistributed in some other biological fluids that can serve as a sample for *in vitro* diagnostic (IVD) tests (2, 9, 56–62).

Tuberculosis (TB) is a good example of an infectious disease for which cfDNA would be especially promising. Of the estimated 10.4 million active TB cases occurring worldwide in 2016, it is estimated that 40% of the cases remained either undiagnosed or unreported, in large part due to inadequate diagnostics (15, 16). Currently, most tests for TB diagnosis require a sputum sample, with sputum microscopy being the most widely used test. Unfortunately, the current sputum-based diagnostics have limited accuracy and have limited applicability in population groups who have difficulty providing sputum (e.g., children, patients with HIV-associated TB, or extrapulmonary TB cases). Most blood-based assays that are in development lack specificity as they rely on host markers (17, 18). cfDNA indicates the presence of the pathogen, and as such, it is an attractive biomarker for TB detection and treatment monitoring of M. tuberculosis for pulmonary as well as extrapulmonary TB in any age group using noninvasive samples, such as urine (4). Figure 1 is a schematic drawing of the origin, release, and potential diagnostic use of M. tuberculosis cfDNA.

At the first World Health Organization Global Ministerial Conference on Ending Tuberculosis, Anthony Fauci and colleagues highlighted that “we need to think about TB in modern terms and use cutting-edge technologies,” in order to start thinking about ending TB (19). cfDNA-based tests have the potential to improve TB diagnosis and be a true “game-changer.” The intention of the manuscript is to (i) review the current evidence and potential of blood and urinary cfDNA as a biomarker for TB, and (ii) describe the main challenges for the development of an appropriate *in vitro* diagnostic (IVD) test for cfDNA-based TB detection for use in limited resource settings.

### The case of tuberculosis.

In this section, we describe the state of the art cfDNA TB detection and treatment monitoring and present the results and limitations of the published literature, as well as the desired characteristics for future studies in this area. A comprehensive search of the literature identified 15 studies that describe the detection of M. tuberculosis DNA in the blood and urine of TB patients with nongenitourinary tuberculosis using nucleic acid amplification.

### Detection in blood.

We identified five studies detecting M. tuberculosis DNA in the blood. Three of these studies assessed peripheral blood mononuclear cells (PBMCs) (7, 20–22) and were not further discussed, as they mainly targeted genomic DNA in the cellular fraction rather than cfDNA (4). The methodological characteristics from these three studies are summarized in Table S1 in the supplemental material. The remaining two studies report the detection of M. tuberculosis cfDNA in plasma, which is considered the blood sample type that provides the most consistent and accurate results for cfDNA analysis (23, 24). Tables 1 and 2 summarizes the accuracy results and main methodological steps used to isolate cfDNA described in these studies. Ushio et al. used digital PCR and reported a sensitivity/specificity of 65%/93% and 29%/100% depending on the threshold set; while Click et al. showed a sensitivity and specificity of 43% and 67%, respectively (23, 24).

**TABLE 1:** cfDNA isolation methodology of studies^*a*^ on blood- and urine-based cfDNA detection of M. tuberculosis by nucleic acid amplification techniques in which the methodological steps are *a priori* considered suitable for cfDNA isolation and detection^*b*^^,^^*c*^

Publication’s first author	Yr	Sample type	Centrifugation, urine supernate collection	Preservative/storage	DNA extraction method	Test type	Target(s)	Amplicon target size(s) (bp)
Ushio	2016	Plasma	NA	EDTA/NR	Qiagen DNeasy blood and tissue kit	Digital PCR	IS6110, gyrB	71
Click	2018	Plasma	NA	EDTA/NR	QiaAmp circulating nucleic acid kit	qPCR	IS6110	106
Cannas	2008	Urine	Yes	EDTA/NR	Manual/resin	Nested PCR	IS6110	67 and 129
Fortun	2014	Urine	NR	NR/NR	NR	TMA	16S rRNA	NR
Labugger	2017	Urine	Yes	EDTA/NR	Manual/resin	PCR	IS6110	38
Patel	2017	Urine	NR	EDTA/NR	Manual/resin	PCR	DR region	38

a See references 23, 24, 25, 32, 33, and 34.

b Two additional studies reported one case report (63, 64). Both studies describe the identification of urinary M. tuberculosis cfDNA in extrapulmonary TB cases; the first refers to a disseminated TB case while the second to a pediatric tubercular otitis media case. Sample preanalytical steps were performed as reported in the Ushio et al. study and Cannas et al. study, respectively (63, 64). Data from these studies were not included here given that only samples from an individual patient were available.

c NR, not reported; NA, not applicable; TMA, transcription-mediated amplification; DR, direct repeat.

**TABLE 2:** Performance estimates of studies^*a*^ on blood- and urine-based cfDNA detection of M. tuberculosis by nucleic acid amplification techniques in which the methodological steps are *a priori* considered suitable for cfDNA isolation and detection^*b*^

Publication first author	Yr	Sample type	TB presentation	HIV positive (%)	Smear positive (%)	Method of TB confirmation	% (no./total no. of samples) of indicating:	
							Sensitivity	Specificity
Ushio	2016	Plasma	Pulmonary	0	100	Culture	65 (21/33)	93 (18/19)
							29 (10/33)	100 (19/19)
Click	2018	Plasma	Pulmonary	64	100	Culture and/or Xpert	45 (18/40)	67 (2/3)
Cannas	2008	Urine	Pulmonary	5	95	Sputum smear or culture	79 (34/43)	100 (23/23)
Fortun	2014	Urine	Pulmonary	12	NR	Culture	18 (5/28)	NR
			Extrapulmonary	29	NR		70 (57/82)	NR
Labugger	2017	Urine	Pulmonary	0	60	Culture	64 (7/11)	100 (8/8)
Patel	2017	Urine	Pulmonary	38	33	Culture	43 (75/175)	89 (210/237)

a See references 23, 24, 25, 32, 33, and 34.

b NR, not reported; Xpert, Xpert MTB/RIF assay.

### Detection in urine.

Regarding urinary cfDNA studies, in 2008 Cannas et al. first demonstrated the presence of short DNA fragments, smaller than 200 bp, of M. tuberculosis-specific DNA in the acellular fraction of urinary samples of pulmonary TB patients from either degraded or metabolically active bacteria that had crossed the kidney barrier (25). Until then, all studies targeting M. tuberculosis in urine performed a centrifugation step, extracted DNA from the urine sediment, and concentrated high-molecular-weight amplicons (approximately 500 bp) (26–31). However, urinary cfDNA is expected to be found in the acellular fraction due to its size (∼170 bp) (3–5), and consequently, these studies primarily detected M. tuberculosis genomic DNA instead of cfDNA. The results reported, therefore, were considered to be irrelevant for this review (25). The methodological characteristics corresponding to these studies are summarized in Table S1.

**FIG 2:**
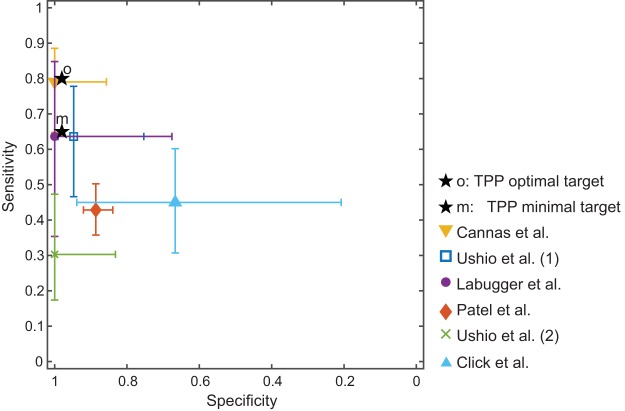
Performance (sensitivity versus specificity) of studies on blood and urine-based cfDNA detection of M. tuberculosis by nucleic acid amplification techniques in which the methodological steps are *a priori* considered suitable for cfDNA isolation and detection. Studies reporting only the sensitivity or specificity are excluded (23–25, 32–34). TPP, target product profile.

In total, we identified only four studies focusing on urinary cfDNA and cfRNA for TB done after 2008 in which the methodological steps seem *a priori* suitable for cfDNA/cfRNA isolation and detection (25, 32–34). The amplicon size in these studies was small, mostly lower than 100 bp, and cfDNA/cfRNA was extracted using the cell-free urinary fraction. A summary of the performance and methodology of these studies is also presented in Tables 1 and 2. Sensitivities and specificities achieved in these studies fall in the range of 43% to 79% and 89% to 100%, respectively, for pulmonary and extrapulmonary TB cases. Figure 2 shows the sensitivities, specificities, and 95% confidence intervals of the plasma and urinary studies which followed *a priori* acceptable methodology for cfDNA/cfRNA isolation. Confidence intervals were calculated according to the Wilson score method (35).

Considering that the TB target product profile (TPP) prioritized by the WHO stipulates a performance of 65%/98% as minimally acceptable and 80%/98% (sensitivity/specificity) as optimal for a nonsputum biomarker detection test (36), the performance results for plasma and urinary cfDNA studies (summarized in Table 2) appear potentially promising for TB detection tests. However, all of these studies have methodological limitations, and the lack of methodological consistency between studies make comparisons almost impossible (7, 37). First, they lack standardized methodology for sample collection, sample storage, and cfDNA extraction, especially in the urinary cfDNA studies, and some of them do not even describe the methodology followed (32, 38). Second, only three studies were performed in high TB burden countries (China, South Africa, and Kenya) (23–25, 32–34). Third, the number of patients enrolled in these studies was small except in the case study of Patel et al. (34). The Patel study enrolled a representative patient population with over 400 pulmonary TB suspects. The study reports a low sensitivity (43%) and moderately high specificity (89%) and proposes its use in combination with smear microscopy since the sensitivity achieved when both tests are used together is considerably higher (83.8%) than for each test alone (smear microscopy alone: 75.1%). This study hypothesizes that the low sensitivity observed could be due to the preanalytical steps followed (34).

Although it used only a few samples, the Labugger et al. study should be mentioned specifically, as it analyzed the correlation between cfDNA and the time to positivity in cultures or acid-fast bacillus (AFB) microscopy scores at the onset of treatment and no correlation was observed (33). However, maximal cfDNA levels correlated well with a radiological score. In this study, the assay results were not correlated with renal function parameters and general inflammatory status shown by the absence of significant correlation between maximal cfDNA and creatinine, urea, and cyclic AMP receptor protein (CRP) levels. (33).

### Determining the cfDNA concentration present.

From the studies summarized in Tables 1 and 2, we aimed to estimate the concentration of M. tuberculosis cfDNA present in plasma and urine. Two of the six studies provided sufficient information (23, 33). Both studies used the repetitive insertion element IS6110 as a target, and the cfDNA concentrations are referred to as IS6110 targets rather than M. tuberculosis genomes. The Labugger et al. study provides concentration values for urinary and blood samples, while the Ushio et al. study focused on blood.

Based on the Labugger et al. study, the median urinary M. tuberculosis cfDNA concentration in treatment-naive patients was 6.3 copies/ml. One week after treatment initiation, the median value rose to 25 copies/ml but decreased to 2.5 copies/ml at week 11. The highest urinary cfDNA value measured in a TB patient was 1,000 copies/ml. The minimum concentration measured with the reported PCR assay was 3 copies/ml (33). In order to achieve high sensitivity given the very low number of copies/ml in urine, the authors collected a large volume of urine (4 ml), and isolated and concentrated the cfDNA in 50 µl. The PCR sample input volume of 100 µl was also large, of which 20 µl was concentrated cfDNA. The plasma concentration reported range was very similar to the urinary cfDNA range (33).

In the Ushio et al. study, the estimated median concentration of M. tuberculosis cfDNA was 110 copies/ml, with mean ± standard deviation (SD) of 7,200 ± 29,150 copies/ml in blood plasma of patients with active TB. These numbers differed significantly from those observed for healthy control subjects, whose median concentration was 0 copies/ml and mean ± SD was 22 ± 24 copies/ml. The minimum concentration measured with the reported digital PCR assay was approximately 50 copies/ml of plasma. The authors extracted cfDNA from 200 µl of plasma, eluted it in 40 µl, and performed PCR using 4 µl of the concentrated cfDNA sample (23).

### Future research necessary.

To shed further light on the potential role of cfDNA for TB diagnosis in the future, well-curated biorepositories are necessary. Samples should be characterized based on a combination of quantitative microbiology from multiple sputum and nonsputum samples (including an assessment of time to positivity on culture and cycle threshold on molecular assays) and ideally also with positron emission tomography (PET) as a way to identify subclinical or incipient TB (39). The biorepositories should collect matched plasma and urine samples. Additionally, reference materials for M. tuberculosis cfDNA are highly needed as quality controls for validating different cfDNA methods and ultimately for clinical management of patients based on absolute standard values (40).

The evaluation of the concentration range of M. tuberculosis cfDNA in biological samples should be an important goal in the TB cfDNA research agenda. Estimating total M. tuberculosis body burden is key not only for diagnosis but also for treatment monitoring (41). cfDNA may identify disease anywhere in the body and potentially detect disease states that are currently not identifiable with classical microbiological methods. The correlation of M. tuberculosis cfDNA concentration with other nonsputum biomarkers (e.g., TB antigens such as lipoarabinomannan) should also be assessed. Preclinical studies assessing novel chemical entities for treatment could be used to calculate terminal CFU/lesion or lung and correlate with immediate premortem cfDNA copies/ml, ideally with PET computed tomography (CT) (a nonhuman primates study), as well as a longitudinal analysis of cfDNA. Estimating the M. tuberculosis cfDNA concentration in biological samples is also salient for assay development, including sample preparation (sample collection volume, cfDNA isolation method, and concentration volume), and for the detection platform development (assay limit of detection). Based on the cfDNA concentrations estimated in plasma and urine samples (median, 6.3 copies/ml) from the Labugger et al. study, we recommend, for a nucleic acid amplification-based test with a limit of detection of 10 copies/µl, collecting at least 5-ml samples and applying a concentration step prior to cfDNA detection. Given the large volume of sample required, urinary collection would be preferred in pediatric patients.

More studies are also required on HIV-positive and pediatric patients. A limited number of studies are currently available for TB cfDNA-based diagnosis of HIV patients, but no studies were identified for pediatric patients. These population groups would be the ones who would benefit the most from a cfDNA-based assay given the difficulty they have to produce sputum. Most studies are also inadequate in that they include a very high proportion of persons with TB (especially smear-positive TB) and few controls. This may limit the ability to extrapolate performance characteristics from these studies to populations that have a much lower prevalence of disease and likely more paucibacillary disease.

Beyond the identification of M. tuberculosis, cfDNA analysis would permit us to gain a deeper understanding of infections. Burnham et al. used sequencing techniques for cfDNA analysis and reported cfDNA for quantification of bacterial growth, identification of antimicrobial resistance genes, and evaluation of the host response to infection on the cellular and tissue level for urinary tract infections (42). The same should be explored for TB. cfDNA analysis also has the potential to assess the response to drug therapy and provide information regarding disease state. Provided detection technologies allow for sufficiently low sensitivity (not possible with current tools), cfDNA could be used to identify incipient TB. The impact of such tests would be substantial. However, it is still unclear whether M. tuberculosis burden, or the burden of its products such as cfDNA, is directly correlated with the progression from latent to active disease (41).

### Challenges in the development of a cfDNA-based IVD test.

In the following section, the main challenges for the development of an IVD test are described. One of the main challenges for the development of a cfDNA assay is performing appropriate sample pretreatment (i.e., sample processing prior to the amplification and detection step), given the low cfDNA concentration in biological fluids. Unfortunately, despite the large number of research studies in the field of cfDNA, there is a lack of standardization in preanalytical methodology according to numerous reviews in the field (7, 37, 43–45). The preanalytical factors which might affect cfDNA concentration and fragmentation include (i) sample collection, (ii) sample storage, (iii) cfDNA extraction, and (iv) storage of cfDNA extracts. Blood is the matrix that has been most widely studied since it is the matrix of choice for cancer and prenatal analyses, but few preanalytical studies are available regarding cfDNA in urine. Table 3 presents a summary of some research gaps identified relating to preanalytical factors for cfDNA. More information about preanalytical factors for cfDNA can be found in studies and reviews about this topic (7, 37, 46–50). To guarantee conclusive results, the standardization of sample collection to ensure uniform preanalytical handling of a sample will be critical. This is of particular importance when pathways from sample collection to processing are long, as is often the case in resource-limited settings. To summarize, universal cfDNA standards and reference materials are needed to harmonize results and methods across time, and they should be made available to test developers and researchers (40).

**TABLE 3:** Research gaps for the development of a cfDNA-based IVD test related to preanalytical factors

Research gaps: lack of standard operating procedures for cfDNA preanalytical steps, especially for urinary samples
Which is the best extraction kit/method for different sample types?
How rapidly do samples need to be processed to avoid degradation with and without preservatives?
Which urine preservative works best to avoid cfDNA degradation?
How long can plasma samples and urinary samples with preservatives be stored?
How long can cfDNA extracts be stored at −20°C and −80°C?

Another challenging feature for the development of a cfDNA assay is integrating sample pretreatment and a detection technology within a single sample-to-answer device. The setting where the test is expected to be used, as well as the concentration of target cfDNA in the sample, will guide the selection of appropriate test platforms. Costly techniques, such as sequencing, digital PCR (dPCR), and mass spectrometry, mostly used in resource-rich contexts have to date limited applicability in low-resource settings, unless cost and complexity can be reduced substantially (as is expected for sequencing) (2). More affordable technologies that are readily applicable and already in use in resource-limited settings, such as PCR, isothermal amplification, or line-probe assays, are better suited. Nucleic acid-detection techniques bypassing amplification and based on hybridization of sequence-specific probes (such as fluorescence *in situ* hybridization [FISH]) are probably unsuitable due to the low concentration of cfDNA in biological samples. Typical limits of detection of these platforms are 0.5 to 5 copies/µl for PCR and >10 copies/reaction for isothermal amplification (51, 52). New diagnostic techniques that are worth mentioning in this context are those based on clustered regularly interspaced short palindromic repeats (CRISPR) technology, such as specific high-sensitivity enzymatic reporter unlocking (SHERLOCK) and DNA endonuclease-targeted CRISPR trans reporter (DETECTR) (53–55). They both use isothermal amplification and Cas-mediated collateral cleavage of a reporter RNA, allowing for detection of the target.

To the best of our knowledge, no cfDNA assays for infectious diseases based on PCR or isothermal amplification, which integrate sample pretreatment, are currently available. Many of the existing nucleic acid tests (NATs) for infectious diseases, which use PCR or isothermal amplification, use a concentration of the pathogen, cell or viral lysis, and an extraction of the genomic material followed by amplification. NATs for cfDNA have different requirements for sample preparation. The main differences include (i) no infectious agent enrichment or cell lysis, (ii) emphasis on enrichment of short DNA fragments instead of genomic DNA, and (iii) amplification of short amplicons.

In summary, the field of cfDNA as a TB biomarker is still in its infancy; however, its high potential is driving more attention to this area. Most papers about this topic were published in the last 3 years and more promising results are expected in the near future. Once cfDNA is validated as a TB biomarker with sufficient performance for diagnosis, more efforts need to be directed toward the development of an affordable platform ideally able to detect this infectious disease at the point of care.

## Supplementary Material

Supplemental file 1
