# Supplementary material for: Toward the Development of a Circulating Free DNA-Based In Vitro Diagnostic Test for Infectious Diseases: a Review of Evidence for Tuberculosis
Source: J Clin Microbiol. 2019 Mar 28;57(4):e01234-18. doi: 10.1128/JCM.01234-18 (PMC6440766; doi:10.1128/JCM.01234-18)
Supplement: Supplemental file 1 [file JCM.01234-18-s0001.pdf]

Supplemental data

SUPPLEMENTARY TABLE 1A cfDNA isolation methodology of studies on blood and urine-based detection of *M. tuberculosis* DNA by nucleic acid amplification techniques in which the methodological steps are considered unsuitable for cfDNA isolation and detection, thus the results reported are considered to be biased (26–31). NR: Not reported; ‡ very probably did not include supernate collection after a centrifugation step since it follows a 1991 protocol in which centrifugation and pellet collection was the standard procedure.

1st publication author	Year	Sample type	Centrifugation, urine supernate collection	Preservative /Storage	DNA extraction method	Test type	Target	Amplicon target size (bp)
Condos	1996	Blood	NA	NR/NR	Proteinase K, RNAse treated; phenol–chloroform extracted	Nested PCR	IS6110	NR
Ahmed	1998	Blood	NA	NR/NR	Proteinase K, RNAse treated; phenol–chloroform extracted	PCR	IS1081	306 bp
Taci	2002	Blood	NA	NR/NR	Manual/Heating(55)	PCR	IS986	245 bp
Sechi	1997	Urine	No	NR/NR	None	Nested PCR	IS6110	182 bp & 566 bp
Aceti	1999	Urine	No	NR/NR	None	Nested PCR	IS6110	309 bp & 566 bp
Kafwabulula	2002	Urine	No	No/NR	Proteinase K, RNAse treated; phenol–chloroform extracted	Nested PCR	IS6110	181 bp & 566 bp
Torrea	2005	Urine	No	NR	Freeze and thawing; phenol– chloroform extraction	Nested PCR	IS6110	309 bp & 566 bp

Rebollo	2006	Urine	NR ‡	NR/NR	NR	PCR	IS6110	123 bp
Gopinath and Singh	2009	Urine	No	No/up to 3 days at 4 °C	Lysozyme and proteinase K treated; phenol–chloroform extracted	PCR	cfp32	786 bp

SUPPLEMENTARY TABLE 1B Performance estimates of studies on blood and urine-based detection of *M. tuberculosis* DNA by nucleic acid amplification techniques in which the methodological steps are considered unsuitable for cfDNA isolation and detection, thus the results reported are considered to be biased (26–31). NR: Not reported.

1st publication author	Year	Sample type	TB presentation	Method of TB confirmation	Sensitivity	Specificity
Condos	1996	Blood	Pulmonary	Culture	95% (39/41)	89% (42/47)
Ahmed	1998	Blood	Pulmonary	Sputum smear and chest X-ray	44% (7/16)	100% (10/10)
Taci	2002	Blood	Pulmonary	Sputum smear	40% (16/40)	100% (20/20)
Sechi	1997	Urine	NR	NR	NR	NR
Aceti	1999	Urine	Pulmonary	Sputum smear or culture	100% (13/13)	93% (1/143)
Kafwabulula	2002	Urine	Pulmonary	Sputum smear or culture	56% (35/63)	98% (62/63)
Torrea	2005	Urine	Pulmonary	Sputum smear or culture	44% (108/247)	98% (54/55)
			Extrapulmonary	Clinical criteria	57% (48/84)	
Rebollo	2006	Urine	Pulmonary, Extrapulmonary, & Disseminated	Culture and response to treatment	16% (9/57)	100% (26/26)
Gopinath and Singh	2009	Urine	Pulmonary	Sputum culture	52% (24/46)	71% (10/35)[#]

[#]The study included 112 controls and reported PCR negative results for all of them.

SUPPLEMENTARY TABLE 2 Commercially available cfDNA sample collection tubes. The aim of this list is not to be comprehensive.

Name	Sample type	Manufacturer
Collection tube	Blood	Norgen Biotek
Collection tube	Blood	Streck
Collection tube	Blood	Quiagen
Collection tube	Blood	Becton Dickinson
Urine conditioning buffer	Urine	ZYMO Research
cfDNA urine preserve	Urine	Streck

SUPPLEMENTARY TABLE 3. Commercially available IVD kits for cfDNA extraction and purification. The aim of this list is not to be comprehensive.

Name	Sample type	Manufacturer	Manual/Automated processing
QIAamp circulating nucleic acid kit	Blood	QIAGEN	Manual
NucleoSpin® Plasma XS kit	Blood	Macherey-Nagel	Manual
FitAmp™ plasma/serum DNA isolation kit	Blood	Epigentek	Manual
Sentosa SX cfDNA kit	Blood	Vela Diagnostics	Automated
Mag-Bind® cfDNA Kit	Blood	Omega Bio-tek	Automated
Maxwell® RSC ccfDNA Plasma Kit	Blood	Promega	Automated
QIAamp circulating nucleic acid kit	Urine	QIAGEN	Manual
Extract-all urine DNA isolation kit	Urine	Zymo Research	Manual
Urine DNA isolation kit slurry format	Urine	Norgen Biotek	Manual
MagMAX™ Cell-Free DNA Isolation Kit	Blood, Urine	Thermo Fisher	Automated
